# A Wide-Band Magnetoelectric Sensor Based on a Negative-Feedback Compensated Readout Circuit

**DOI:** 10.3390/s24020423

**Published:** 2024-01-10

**Authors:** Yang Qiu, Lingshan Shi, Longyu Chen, Yuxuan Yu, Guoliang Yu, Mingmin Zhu, Haomiao Zhou

**Affiliations:** The Key Laboratory of Electromagnetic Wave Information Technology and Metrology of Zhejiang Province, College of Information Engineering, China Jiliang University, Hangzhou 310018, China; qiuyang@cjlu.edu.cn (Y.Q.); lingshanshi@cjlu.edu.cn (L.S.); 2100310101@cjlu.edu.cn (L.C.); 2100310113@cjlu.edu.cn (Y.Y.); glyu@cjlu.edu.cn (G.Y.); mzhu@cjlu.edu.cn (M.Z.)

**Keywords:** magnetoelectric sensor, negative-feedback readout circuit, bandwidth

## Abstract

Magnetoelectric (ME) sensors cannot effectively detect broadband magnetic field signals due to their narrow bandwidth, and existing readout circuits are unable to vary the bandwidth of the sensors. To expand the bandwidth, this paper introduces a negative-feedback readout circuit, fabricated by introducing a negative-feedback compensation circuit based on the direct readout circuit of the ME sensor. The negative-feedback compensation circuit contains a current amplifier, a feedback resistor, and a feedback coil. For this purpose, a Metglas/PVDF/Metglas ME sensor was prepared. Experimental measurements show that there is a six-fold difference between the maximum and minimum values of the ME voltage coefficients in the 6–39 kHz frequency band for the ME sensor without the negative-feedback compensation circuit when the sensor operates at the optimal bias magnetic field. However, the ME voltage coefficient in this band remains stable, at 900 V/T, after the charge amplification of the direct-reading circuit and the negative-feedback circuit. In addition, experimental results show that this negative-feedback readout circuit does not increase the equivalent magnetic noise of the sensor, with a noise level of 240 pT/√Hz in the frequency band lower than 25 kHz, 63 pT/√Hz around the resonance frequency of 30 kHz, and 620 pT/√Hz at 39 kHz. This paper proposes a negative-feedback readout circuit based on the direct readout circuit, which greatly increases the bandwidth of ME sensors and promotes the widespread application of ME sensors in the fields of broadband weak magnetic signal detection and DBS electrode positioning.

## 1. Introduction

Magnetoelectric (ME) composites, comprising magnetostrictive materials and piezoelectric materials, have gained significant attention in recent years owing to their strong ME effect. As magnetic field sensors, they can be used to detect low-frequency (0–100 Hz) magnetic fields; further, their response at the resonant frequency is particularly advantageous, and they are expected to surpass conventional high-performance weak magnetic sensors in terms of sensitivity, bandwidth, power consumption, size, cost, and other comprehensive indexes [1,2,3,4,5]. Several studies have also focused on further improving the magnetic field detection capability and performance of ME sensors at low frequencies (0–100 Hz) and resonant frequencies [6,7,8,9,10,11]. For example, Y. Shen et al. prepared a Metglas/PZT ME sensor capable of detecting DC magnetic fields as low as 1 nT, which can be used for geomagnetic measurements [6]. Z. Chu et al. proposed an amplitude modulation method (AMM) for quasi-static magnetic field detection with a Metglas/PMN-PT ME sensor and lock-in amplifier, capable of detecting 100 mHz AC magnetic fields down to 100 pT; their proposed method makes an important contribution to the application of magnetoelectric sensors in the field of detecting very-low-frequency signals [7]. P. Hayes et al. used electric field modulation on a PZT/FeCoSiB/AlN sensor to detect low-frequency magnetic fields; their method not only overcomes the disadvantages of the frequency conversion method in terms of energy consumption and frequency conversion crosstalk but also gives the sensor an equivalent magnetic noise of 10 nT/√Hz at 10 Hz [12]. S. Zabe et al. prepared the AlN/FeCoSiB sensor, which operates based on the delta-E effect with a noise level of approximately 100 pT/√Hz within a bandwidth of 100 Hz [13]. C. Dong et al. developed a very low frequency (VLF) communication system based on a Metglas/PZT magnetoelectric antenna; the sensor had an equivalent magnetic noise of 240 fT/√Hz at the resonant frequency of 23.95 kHz, which has great potential for application in the field of low-frequency communication [8]. A. V. Turutin et al. prepared a magnetoelectric Metglas/bidomain y +140°-cut lithium niobate composite, capable of achieving an equivalent magnetic noise of 92 fT/√Hz at a resonant frequency of 6.862 kHz, which is expected to be applied in the field of biomedicine [9].

Although ME sensors exhibit excellent performance in terms of magnetic field detection in DC magnetic fields at low and resonant frequencies, when detecting AC magnetic fields, the ME voltage coefficient of the sensor itself varies very significantly with frequency. This occurs especially at resonant frequencies, where the ME voltage coefficient is significantly enhanced and the output response is several times higher than that at non-resonant frequencies [11,14,15]. The bandwidth of a resonant ME sensor is normally below 1 kHz due to the high mechanical quality factor, which is a major limitation facing practical engineering applications [16]. In fact, a certain bandwidth for the magnetic sensor is usually required for practical applications; i.e., the ME voltage coefficient should be maintained as constant as possible in the given frequency range to measure the magnetic field signal without distortion. For example, in ultra-low-field magnetic resonance imaging (MRI) systems, the MRI signal is generally distributed between 1 kHz and 10 kHz because the main magnetic field B_0_ is of the order of μT [17]. The deep brain stimulation (DBS) therapy system is crucial for the treatment of Parkinson’s disease. The precise positioning of its stimulation tool—the DBS electrode—at the location of the brain can effectively improve the treatment effect; it has been found that ME sensors could potentially be used for the localization of DBS electrodes, with a required bandwidth of 10 kHz–85 kHz for the localization signal [18]. Geophysical measurements using transient electromagnetics (TEM) methods require the extension of the readout bandwidth of the sensor to 5 MHz [19]. In the past, there has been research on using readout circuits to change the operating frequency band of magnetoelectric sensors. The modulated readout circuit applies an alternating magnetic field of a known amplitude and frequency to the ME sensor, which can help modulate the operating frequency band of the sensor to the DC magnetic field and low-frequency magnetic field [6,7]. The direct readout circuit only involves a simple charge amplification of the ME sensor output signal, which does not change the operating frequency band of the sensor and is mainly used for magnetic field measurements at the resonant frequency points [20,21]. Therefore, none of the aforementioned circuits can achieve wide-band magnetic field detection. V. Serov et al. introduced a negative-feedback circuit containing a current amplifier, a voltage divider, and a compensation coil into the magnetoelectric sensor readout circuit to extend the linear measurement range of the sensor for DC magnetic fields [22]; however, this research did not focus on the operating bandwidth of the magnetoelectric sensor.

In this paper, ME sensors were prepared using Metglas without the need for pre-stress and PVDF with good flexibility. Using the concept of negative feedback, a negative-feedback circuit was introduced on the basis of the direct readout circuit, such that the ME voltage coefficient of the ME sensor and its readout circuit depended mainly on the negative feedback loop. This enabled the ME voltage coefficient in the 6–39 kHz band to be stabilized at 900 V/T, increasing the bandwidth of the ME sensor and allowing it to measure signals with a certain bandwidth without distortion. Further, this method did not introduce additional noise, with an equivalent magnetic noise of 240 pT/√Hz at less than 25 kHz. This readout circuit can overcome the limitations of modulated readout circuits and direct readout circuits, which have a narrow bandwidth, and lay the foundation for future applications of magnetoelectric sensors in biomagnetic measurements, DBS electrode positioning, TEM, and other fields.

## 2. Preparation and Performance Characterization of the ME Sensor

**Figure 1:**
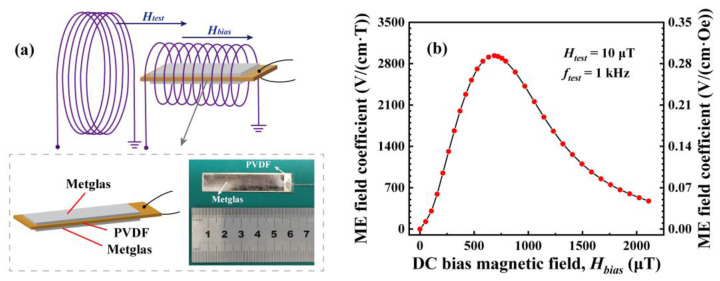
(**a**) Schematic and physical diagram of the structure of the ME sensor; (**b**) the ME field coefficient as a function of the DC bias magnetic field *H_bias_* at *H_test_* = 10 μT, *f_test_* = 1 kHz.

A photograph of the ME composite sensor fabricated in this work is shown in the dashed box in Figure 1a. It contained PVDF (Measurement Specialties, Fairfield, NJ, USA) with dimensions of 12 mm × 55 mm × 52 μm, poled along the thickness direction (piezoelectric coefficient *d*_33_ = −33 pC/N), and Metglas (RA0202MG, Advanced Technology & Materials Co., Ltd., Beijing, China) with dimensions of 10 mm × 50 mm × 26 μm, magnetized along the longitudinal direction (magnetostrictive coefficient *λ* = 27 ppm). A layer of Metglas was bonded onto both the top and bottom surfaces of the PVDF layer using an epoxy resin (CC-33A, KYOWA, Japan), which was then cured at room temperature for 24 h in a vacuum bag. The output of the response voltage of the ME composite was then achieved by gluing copper wires to the silver electrode surfaces on the top and bottom sides of the PVDF using silver adhesive.

We applied the AC test magnetic field *H_test_* at 1 kHz through a test coil with a diameter of 0.2 m, 280 turns, a length of 140 mm, and a resistance of 12.8 Ω; the DC bias field *H_bias_* was applied through a coil with a diameter of 0.07 m, 210 turns, and a resistance of 6.4 Ω. The axes of the two coils overlap and their axis directions are aligned with the length direction of the sensor; the sensor is placed at the center of the two coils, as shown in Figure 1a. The measurement results of the magnetoelectric coefficient of the sensor are shown in Figure 1b. The ME field coefficient (*α_ME_* = *V_out_*/(*t·H_test_*)) increases and then decreases with increases in *H_bias_*, and the magnetoelectric field coefficient of the sensor reaches its maximum value, of 2936 V/(cm·T), at *H_bias_* = 686 μT. As can be seen in Figure 1b, the ME field coefficient at zero bias is very low and, therefore, the ME sensor was selected to operate in the optimum bias field.

**Figure 2:**
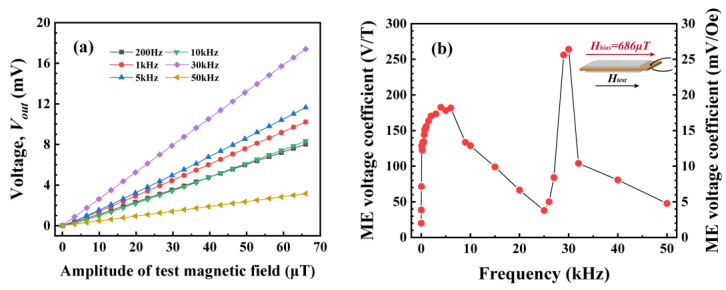
(**a**) Curve of the output voltage *V_out_* of the ME sensor at different frequencies as a function of test magnetic field *H_test_* at *H_bias_* = 686 μT; (**b**) the ME voltage coefficient at different frequencies under the optimal bias magnetic field. The inset indicates the direction of *H_bias_* and *H_test_*.

As shown in Figure 2a, the output voltage of the sensor maintains a good linear relationship with the test magnetic field at *H_bias_* = 686 μT. However, the output voltage of the sensor changes significantly when the amplitude of *H_test_* is kept constant and the frequency is varied. This is evident from ME voltage coefficients (*S* = *V_out_*/*H_test_*) at different frequencies in Figure 2b, where both *H_bias_* and *H_test_* are applied along the length of the sensor, as shown in the inset. It can also be seen from Figure 2b that the variation in the ME voltage coefficient is large over the entire test frequency range. In the low-frequency range of less than 10 kHz, the ME voltage coefficient of the sensor tends to increase and then decrease, reaching a maximum value of 182 V/T near 4 kHz. However, in the high-frequency band from 10 kHz to 50 kHz, there is a significant upward and then downward trend, especially near the resonant frequency of 30 kHz, where the ME voltage coefficient is as high as 264 V/T; this is much higher than 122.4 V/T at 200 Hz and 47.7 V/T at 501 kHz, and the difference is obvious. From the above results, it can be seen that the ME sensor is suitable for single-frequency weak magnetic signal detection; however, further improvements are required if it is to be directly applied to the detection of weak magnetic signals that require a certain frequency bandwidth.

For this reason, this paper introduces a negative-feedback compensation circuit in the direct readout circuit so that the ME voltage coefficients of the ME sensor can have better linearization with frequency and, thus, expand the operating bandwidth. Further, the use of a negative-feedback compensation circuit does not introduce additional noise.

## 3. Negative-Feedback Readout Circuit

**Figure 3:**
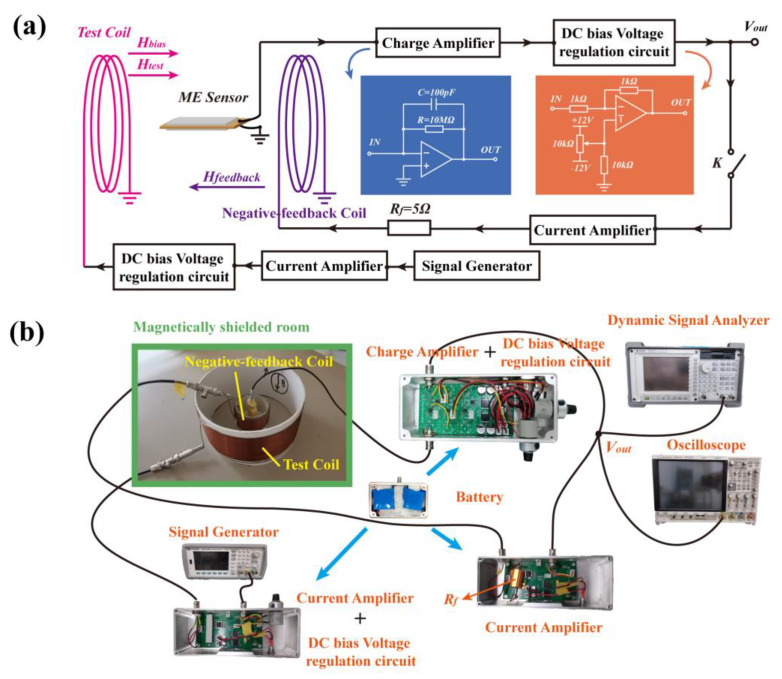
(**a**) Schematic of the negative-feedback readout circuit; (**b**) photo of the setup.

To increase the operating bandwidth of the ME sensor, we designed a direct readout circuit with a negative-feedback compensation circuit to increase the linearization range of the ME voltage coefficient and, thus, increase the bandwidth of the ME sensor. Figure 3a shows a schematic of the negative-feedback readout circuit. To further illustrate the role of the negative-feedback readout circuit in magnetic signal testing, we tested the performance of the system in both the open-loop and closed-loop cases. The test process was performed in a magnetically shielded room located in the Shanghai Institute of Microsystem and Information Technology, Chinese Academy of Sciences. The performance of the magnetically shielded room is 37 dB@0.1 Hz, 52 dB@1 Hz, and 73 dB@10 Hz, which can greatly reduce the interference of magnetic fields in the environment. The test system is shown in Figure 3b. The signal generator (33500B, Agilent, Santa Clara, CA, USA) is connected to the coil through a current amplifier based on the OPA547 chip, and AC signals with different frequencies (200 Hz–50 kHz) are fed to the test coil to generate a test magnetic field *H_test_* with an amplitude of 0–66 μT. The current amplifier can amplify the signal four-fold and is used to adjust the bias voltage. To reduce the interference between the coils and the number of coils, the AC test coil and the DC bias coil—shown in Figure 1a —are combined into one coil; therefore, both *H_test_* and *H_bias_* are present in the test coil in Figure 3a. The output of the ME sensor is connected to a direct readout circuit, including a charge amplifier and a bias voltage regulation circuit. The charge amplifier uses a low-current-noise chip, AD795, which has a low current noise of 0.6 fA/√Hz at 1 kHz. The charge amplifier charge gain (Gain) can be calculated using Equation (1), and the high-pass filter cutting frequency *f_HPF_* can be calculated using Equation (2) [23]:

(1) Gain=1/C(mV/C)

(2) $$f_{HPF}=1/\left(2\pi RC\right)$$

The capacitance *C* and resistance *R* are 100 pF and 10 MΩ, respectively, and the theoretical amplification is 10^10^, with a high-pass filter cutting frequency of 159.1 Hz.

The output of the charge amplifier was connected to the bias voltage regulation circuit. An oscilloscope (DSO-X 4034A, Agilent Technologies, Agilent, Santa Clara, CA, USA) and a dynamic signal analyzer (35670A, Agilent, USA) were connected to the output of the bias voltage regulation circuit to observe and measure the amplitude and phase of the signal.

In addition, the bias voltage regulation circuit was connected to the negative-feedback circuit through a negative-feedback switch *K*. The negative-feedback circuit contained a current amplifier circuit based on the OPA547 chip, a feedback resistor *R_f_*, and a feedback coil. The current amplifier circuit used inverting amplification to produce a maximum current of 750 mA, which was sufficient to meet the current required to generate the feedback magnetic field in the feedback coil. The current amplifier was connected to the feedback coil, with a diameter of 0.07 m, 210 turns, and a resistance of 6.4 Ω via the feedback resistor *R_f_* = 5 Ω, to produce a negative-feedback field from 0 to 66 μT in the direction opposite to *H_test_*.

## 4. Results and Discussion

### 4.1. Bandwidth of the ME Sensor

**Figure 4:**
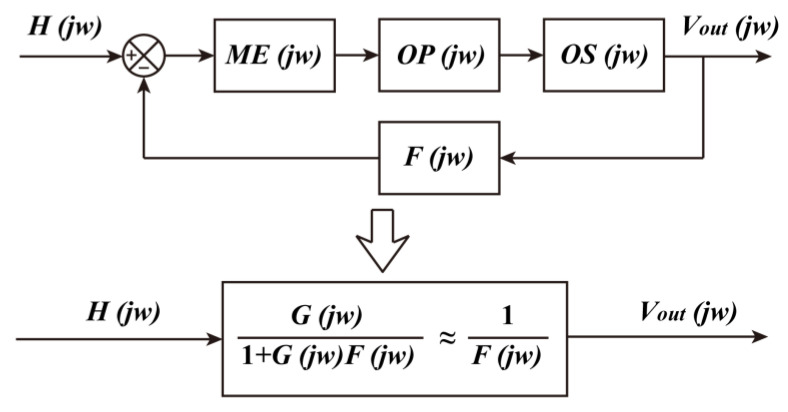
Flow diagram of the negative-feedback readout circuit.

**Figure 5:**
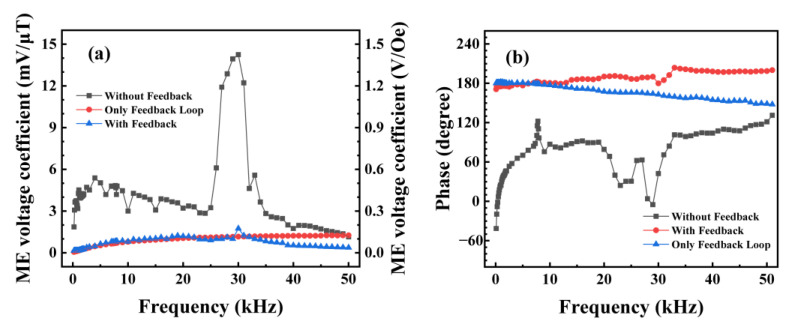
(**a**) ME voltage coefficient and (**b**) phase of the direct readout circuit, the feedback loop, and the negative-feedback readout circuit at different frequencies under the optimal bias magnetic field.

A flow diagram of the negative-feedback readout circuit is shown in Figure 4. The transfer functions of the ME sensor, the charge amplifier, and the bias voltage regulation circuit that form the direct readout circuit are *ME*(*jw*), *OP*(*jw*), and *OS*(*jw*), respectively. When the switch, *K*, is off, the ME sensor outputs in the classical direct readout mode. The input of the ME sensor and its readout circuit is the applied magnetic field signal *H*(*jw*), and the output is the voltage signal *V_out_*(*jw*), whose transfer function is *G*(*jw*) = *V_out_*(*jw*)/*H*(*jw*) = *ME*(*jw*) × *OP*(*jw*) × *OS*(*jw*). The real component of *G*(*jw*), i.e., the ME voltage coefficient of the direct readout circuit, varies with frequency, as shown in the black curve in Figure 5a. The imaginary component of *G*(*jw*), i.e., the phase difference between the input and output signals of the direct readout circuit, varies with frequency, as shown in the black curve in Figure 5b. When the switch, *K*, is off, the signal generator applies a sine wave *V_in_*(*jw*) with different frequencies and an amplitude of 10 mV to the negative-feedback circuit sequentially, testing the magnetic field *H_f_*(*jw*) generated by the feedback coil to obtain the transfer function *F*(*jw*) = *H_f_*(*jw*)/*V_in_*(*jw*) of the entire feedback loop. The reciprocal of this transfer function is calculated 1/*F*(*jw*) = *V_in_*(*jw*)/*H_f_*(*jw*), whose real and imaginary components are shown in the red curve in Figure 5a and 5b, respectively.

When the switch, *K*, is on, the change in charge ΔC+ on the ME sensor caused by the external flux will be compensated by the feedback magnetic field, which is the product of *V_out_*/*R_f_* (feedback current *I_f_*) and *M_f_* (mutual inductance *L_f_* between the ME sensor and the feedback coil). In this switch state, the direct readout circuit and the negative-feedback loop constitute a negative-feedback readout circuit, whose flow diagram is shown in Figure 4. The transfer function of the entire circuit is *G′*(*jw*) = *G*(*jw*)/(1 + *G*(*jw*)*F*(*jw*)). When *G*(*jw*)*F*(*jw*) << 1—i.e., the black curve in Figure 5a is much larger than the red curve—*G*′(*jw*) = 1/*F*(*jw*); the transfer function *G*′(*jw*) of the entire circuit depends mainly on the feedback loop. The real and imaginary components of *G*′(*jw*) are represented by the blue curves in Figure 5a and 5b, respectively.

From the black curve in Figure 5a, it can be seen that the magnetoelectric voltage coefficients of the sensor and direct reading circuit vary significantly. On the whole, the ME voltage coefficient of the sensor shows a trend of increasing first and then decreasing. In the range of 6–25 kHz, the magnetoelectric coefficient is about 3–6 kV/T. In the 25–39 kHz band, the ME voltage coefficient changes abruptly, reaching a maximum value of 14.242 kV/T near the resonant frequency of 30 kHz; at 39 kHz, there is a minimum value of 2.019 kV/T, which is 0.176 times (−16.97 dB) the maximum value. With the negative-feedback circuit, the consistency of the ME voltage coefficient in the 6–39 kHz band is greatly optimized, represented by the blue curve in Figure 5a. Except at the resonant frequency of 30.016 kHz, the maximum value is 1.027 kV/T, and the minimum value is 0.726 kV/T, with the minimum being 0.707 times (−3.01 dB) the maximum. The −3dB bandwidth of the ME sensor and its readout circuit around the resonance frequency is 27.0–31.0 kHz. With the addition of a negative feedback compensation circuit, the −3dB bandwidth extends to 6.0–39.0 kHz. Further, in the 39–50 kHz band, the ME voltage coefficient decreases more significantly because the ME sensor and the direct readout circuit are close to the feedback circuit; i.e., the black curve in Figure 5a is close to the red curve, which does not satisfy the condition *G*(*jw*)*F*(*jw*) << 1.

As can be seen from the black curve in Figure 5b, the phase of the sensor and direct readout circuit fluctuates significantly with the frequency range between −45° and 135°. However, as shown in the blue curve in Figure 5b, the phase changes slowly between 140° and 180° with the negative-feedback circuit in the 6–39 kHz band, which reflects a significant improvement in performance.

It is clear that, after the incorporation of the negative-feedback circuit based on the direct reading circuit, the ME voltage coefficient becomes very smooth in the 5–40 kHz band in comparison to the original circuit, which greatly increases the system bandwidth. Therefore, the output voltage of the negative-feedback readout circuit can be directly divided by a fixed ME voltage coefficient, to obtain a distortion-free magnetic field signal in practical applications. This feature makes it possible to apply ME sensors to biomagnetic measurements, DBS electrode positioning, TEM, and other fields.

### 4.2. Equivalent Magnetic Noise of the ME Sensor

**Figure 6:**
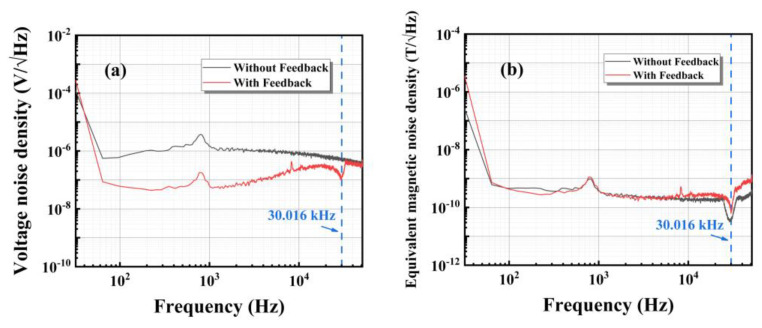
(**a**) Voltage noise density and (**b**) equivalent magnetic noise density of the ME sensor in the openloop and the closed-loop cases.

From the perspective of practical applications, the usefulness of a magnetic sensor depends not only on the response of the sensor to a magnetic field but also on the equivalent magnetic noise generated in the absence of a magnetic field [3]. P. Durdaut et al. demonstrated theoretically that the equivalent noise of surface acoustic wave (SAW) sensors is the same in the open-loop and closed-loop cases [24]. As depicted in Figure 6a, in the absence of a feedback circuit, the ME sensor prepared in this study underwent amplification through a charge amplifier, resulting in an output voltage noise of approximately 870 nV/√Hz at 10 kHz. Upon the integration of a feedback circuit, the ME sensor, prepared in a similar manner, underwent further amplification through the readout circuit, yielding an output voltage noise of about 200 nV/√Hz at 10 kHz. The equivalent magnetic noise in the open-loop and the closed-loop cases was tested without the AC signal given by the signal generator and with the DC bias field retained; the results are shown in the black and red curve in Figure 6b. From the black curve in the figure, it can be observed that the ME sensor and its readout circuit exhibit the minimum equivalent magnetic noise around the resonance frequency of approximately 30 kHz, measuring about 33 pT/√Hz. Within the frequency range of 0.1–39 kHz, excluding the region near the resonance frequency, the equivalent magnetic noise is approximately 240 pT/√Hz. As depicted by the red curve in the figure, the introduction of a feedback compensation circuit results in a noise level of 240 pT/√Hz when the frequency is less than 25 kHz, which is similar to the noise level in the open-loop configuration. Beyond 25 kHz, the equivalent magnetic noise shows a decreasing-then-increasing trend, particularly around the resonance frequency, where the equivalent magnetic noise is approximately 63 pT/√Hz. Subsequently, beyond 30 kHz, the equivalent magnetic noise increases to 620 pT/√Hz at 39 kHz and eventually rises to 900 pT/√Hz at 50 kHz. As can be seen in the figure, the equivalent magnetic noises of the open-loop and the closed-loop case are almost the same under the optimal bias magnetic field. This is because the equivalent magnetic noise sources for both the open-loop and closed-loop cases are ME sensors, so the measured equivalent magnetic noise is essentially the same in the absence of the AC test field. This also indicates that the feedback circuit does not introduce additional noise in the system. In future research, we will optimize the circuit structure by analyzing the noise composition as well as optimizing the structural design of the ME sensor to further improve performance, which will benefit its practical application in broadband weak magnetic signal detection.

## 5. Conclusions

In this paper, a negative-feedback readout circuit for ME sensors is proposed, which utilizes the concept of negative feedback. It is based on the direct readout circuit of the ME sensor, introducing a negative-feedback compensation circuit containing a current amplifier, a feedback resistor, and a feedback coil. The ME sensor unit is made of a Metglas/PVDF/Metglas laminated composite, which operates in the optimal bias magnetic field. To demonstrate the performance of the negative-feedback readout circuit, we first tested its effect on the bandwidth of the magnetoelectric sensor. The experimental results show that the ME voltage coefficient of the ME sensor fluctuates significantly with frequency when there is no negative-feedback circuit, and the difference between the maximum and minimum values in the 6–39 kHz band is more than six-fold. However, in the presence of a negative-feedback circuit, the ME voltage coefficient of the entire negative-feedback readout circuit depends mainly on the negative-feedback circuit, so the ME voltage coefficient in this frequency band is basically stable at 900 V/T. Then, we tested the effect of this readout circuit on the equivalent magnetic noise. The results show that this readout circuit does not introduce additional noise, and the sensor maintains a noise level of 240 pT/√Hz at lower than 25 kHz. The readout circuit proposed in this paper not only increases the bandwidth of the ME sensor but also maintains the equivalent magnetic noise level of the ME sensor, which is significantly superior to that of the existing ME sensor readout circuit. To improve the applicability of ME sensors in fields such as broadband weak magnetic signal detection, future studies should focus on optimizing the equivalent magnetic noise of ME sensors so that they have excellent performance in terms of both broadband width and low noise levels.

## Data Availability

Data are contained within the article.
